# Longitudinal trajectories of anterior cingulate glutamate and subclinical psychotic experiences in early adolescence: the impact of bullying victimization

**DOI:** 10.1038/s41380-023-02382-8

**Published:** 2024-01-05

**Authors:** Naohiro Okada, Noriaki Yahata, Daisuke Koshiyama, Kentaro Morita, Kingo Sawada, Sho Kanata, Shinya Fujikawa, Noriko Sugimoto, Rie Toriyama, Mio Masaoka, Shinsuke Koike, Tsuyoshi Araki, Yukiko Kano, Kaori Endo, Syudo Yamasaki, Shuntaro Ando, Atsushi Nishida, Mariko Hiraiwa-Hasegawa, Richard A. E. Edden, Akira Sawa, Kiyoto Kasai

**Affiliations:** 1 Department of Neuropsychiatry, Graduate School of Medicine, The University of Tokyo, Hongo 7-3-1, Bunkyo-ku, Tokyo 113-8655 Japan; 2 International Research Center for Neurointelligence (WPI-IRCN), The University of Tokyo Institutes for Advanced Study (UTIAS), The University of Tokyo, Hongo 7-3-1, Bunkyo-ku, Tokyo 113-0033 Japan; 3 Institute for Quantum Life Science, National Institutes for Quantum and Radiological Science and Technology, Anagawa 4-9-1, Inage-ku, Chiba, Chiba 263-8555 Japan; 4 Department of Molecular Imaging and Theranostics, Institute for Quantum Medical Science, National Institutes for Quantum Science and Technology, Anagawa 4-9-1, Inage-ku, Chiba, Chiba 263-8555 Japan; 5 Center for Research on Counseling and Support Services, The University of Tokyo, Hongo 7-3-1, Bunkyo-ku, Tokyo 113-0033 Japan; 6 Department of Psychiatry, Teikyo University School of Medicine, Kaga 2-11-1, Itabashi-ku, Tokyo 173-8605 Japan; 7 The University of Tokyo Institute for Diversity and Adaptation of Human Mind (UTIDAHM), The University of Tokyo, Komaba 3-8-1, Meguro-ku, Tokyo 153-8902 Japan; 8 Department of Psychiatry, Teikyo University Mizonokuchi Hospital, Futago 5-1-1, Takatsu-ku, Kawasaki, Kanagawa 213-8507 Japan; 9 Department Child Neuropsychiatry, Graduate School of Medicine, The University of Tokyo, Hongo 7-3-1, Bunkyo-ku, Tokyo 113-8655 Japan; 10 Research Center for Social Science & Medicine, Tokyo Metropolitan Institute of Medical Science, Kamikitazawa 2-1-6, Setagaya-ku, Tokyo 156-8506 Japan; 11 Department of Evolutionary Studies of Biosystems, School of Advanced Sciences, The Graduate University for Advanced Studies (SOKENDAI), Shonan Village, Hayama, Kanagawa 240-0193 Japan; 12 Russell H. Morgan Department of Radiology and Radiological Science, The Johns Hopkins University School of Medicine, 600 N Wolfe St, Baltimore, MD 21287 USA; 13 F. M. Kirby Center for Functional Brain Imaging, Kennedy Krieger Institute, 707 N Broadway Street, Baltimore, MD 21205 USA; 14 Departments of Psychiatry, Neuroscience, Biomedical Engineering, Genetic Medicine, and Pharmacology, Johns Hopkins University School of Medicine, 600 N Wolfe St, Baltimore, MD 21287 USA; 15 Department of Mental Health, Johns Hopkins University Bloomberg School of Public Health, 600 N Wolfe St, Baltimore, MD 21287 USA

**Keywords:** Neuroscience, Psychology, Schizophrenia

## Abstract

Previous studies reported decreased glutamate levels in the anterior cingulate cortex (ACC) in non-treatment-resistant schizophrenia and first-episode psychosis. However, ACC glutamatergic changes in subjects at high-risk for psychosis, and the effects of commonly experienced environmental emotional/social stressors on glutamatergic function in adolescents remain unclear. In this study, adolescents recruited from the general population underwent proton magnetic resonance spectroscopy (MRS) of the pregenual ACC using a 3-Tesla scanner. We explored longitudinal data on the association of combined glutamate-glutamine (Glx) levels, measured by MRS, with subclinical psychotic experiences. Moreover, we investigated associations of bullying victimization, a risk factor for subclinical psychotic experiences, and help-seeking intentions, a coping strategy against stressors including bullying victimization, with Glx levels. Finally, path analyses were conducted to explore multivariate associations. For a contrast analysis, gamma-aminobutyric acid plus macromolecule (GABA+) levels were also analyzed. Negative associations were found between Glx levels and subclinical psychotic experiences at both Times 1 (*n* = 219, mean age 11.5 y) and 2 (*n* = 211, mean age 13.6 y), as well as for over-time changes (*n* = 157, mean interval 2.0 y). Moreover, effects of bullying victimization and bullying victimization × help-seeking intention interaction effects on Glx levels were found (*n* = 156). Specifically, bullying victimization decreased Glx levels, whereas help-seeking intention increased Glx levels only in bullied adolescents. Finally, associations among bullying victimization, help-seeking intention, Glx levels, and subclinical psychotic experiences were revealed. GABA+ analysis revealed no significant results. This is the first adolescent study to reveal longitudinal trajectories of the association between glutamatergic function and subclinical psychotic experiences and to elucidate the effect of commonly experienced environmental emotional/social stressors on glutamatergic function. Our findings may deepen the understanding of how environmental emotional/social stressors induce impaired glutamatergic neurotransmission that could be the underpinning of liability for psychotic experiences in early adolescence.

## Introduction

Schizophrenia is a major psychiatric disorder characterized by positive (hallucinations and delusions), negative (blunted affect, social withdrawal, and anhedonia), and cognitive symptoms. The two major theories for the pathology of schizophrenia are the dopamine hypothesis and the glutamate hypothesis. The dopamine hypothesis can explain the pathology of positive symptoms, because dopamine 2 (D2) receptor (D2DR) antagonists reduce the severity of positive symptoms. However, D2DR antagonists do not improve negative or cognitive symptoms. Since N-methyl-D-aspartate (NMDA) glutamate receptor antagonists such as phencyclidine, ketamine, and dizocilpine induce positive, negative, and cognitive symptoms, the glutamate hypothesis has been established [1, 2]. The causes of NMDA receptor hypofunction in schizophrenia are thought to be varied, including reduced levels of co-agonists such as D-serine and glycine, increased antagonist levels, and reduced NMDA channel expression and trafficking [3]. In addition, it should be noted that, even if none of these possible causes exist, decreased synaptic levels of glutamate would theoretically result in receptor hypofunction because the NMDA receptor is a glutamate receptor. NMDA receptor hypofunction in schizophrenia spectrum disorders has been revealed by genetic [4, 5], neuroimaging (e.g., magnetic resonance [MR] spectroscopy [MRS], positron emission tomography [PET], and single photon emission computed tomography) [6–8], neurophysiological (e.g., mismatch negativity) [9], postmortem [10, 11], and animal studies [12, 13]. In addition, glutamate modulating treatments for schizophrenia have been explored [14]. However, so far, there is limited support from neuroimaging studies including MRS for the hypothesis that glutamatergic abnormalities may be a major neural substrate of schizophrenia [2].

The anterior cingulate cortex (ACC) plays an important role not only in higher cognitive function, including working memory and decision making [15, 16], but also in adaptation to complexity and uncertainty [17]. Additionally, the ACC serves as part of the limbic system and is involved in emotional processing [18, 19]. Furthermore, the ACC is involved in social cognition and sociality [20, 21]. The ACC is subdivided into dorsal and ventral areas, which are mainly involved in cognitive function and emotional processing, respectively [22]. Psychiatric disorders, including schizophrenia, can impair various aspects of psychological function. Several previous MR imaging (MRI) studies have reported structural and functional changes in the ACC in schizophrenia spectrum disorders, including first-episode psychosis (FEP). For example, a meta-analytic study revealed that gray matter volume decreased in the dorsal and ventral ACC in FEP [23]. In addition, a meta-analytic resting-state functional MRI (fMRI) study reported the default mode network’s within-network hypoconnectivity, including the ventral ACC, in FEP [24]. As for high-risk for psychosis, less cortical gray matter in the ACC was found in individuals at clinical high-risk who later transitioned to psychosis [25]. According to a recent fMRI study, ACC functional connectome is implicated in clinical high risk for psychosis [26].

Several recent MRS meta-analytic studies have reported decreases or increases in ACC glutamate levels in schizophrenia spectrum disorders, and the change direction seems to differ according to clinical staging. Glutamate and combined glutamate-glutamine (Glx) levels in the medial frontal cortex and ACC are lower in schizophrenia [27] and glutamate levels in the dorsal ACC are lower in non-treatment-resistant schizophrenia [28], whereas Glx and glutamate levels in the dorsal ACC are higher in treatment-resistant schizophrenia [28]. This discrepancy based on different treatment responses may be linked to the finding from a recent meta-analysis that higher-than-normal glutamate variability in patients with schizophrenia is likely to be found in older subjects [29]. In addition, higher Glx levels in the dorsal ACC and structural changes, such as lower cortical thickness and higher mean diffusivity, in some ACC components were associated in patients with treatment-resistant schizophrenia [30]. The results of ACC glutamatergic levels in FEP were controversial until several years ago [31], whereas recent 7-tesla MRS studies revealed lower glutamate levels in the dorsal ACC in patients with FEP [32, 33]. Some meta-analytic studies reported higher frontal and ACC Glx levels in individuals who are at increased genetic [7] or clinical risk of developing psychosis [34], while another meta-analytic study revealed no significant changes in frontal glutamate or Glx levels in clinical high-risk or genetically high-risk groups [35]. As for associations of glutamatergic function with clinical characteristics, Griffiths et al. reported that ACC glutamate and Glx levels were positively associated with memory function in patients with schizophrenia [36]. Another recent study reported that dorsal ACC Glx levels were negatively associated with psychotic symptoms and positively associated with spatial executive function in ultra-high-risk patients [37]. Demro et al. revealed a positive correlation between ACC glutamate levels and grandiosity in adolescents at increased clinical risk for psychosis [38]. In summary, while results regarding ACC glutamate alterations in individuals at high-risk for psychosis are not yet completely conclusive and patients with treatment-resistant schizophrenia demonstrate higher glutamate levels in the dorsal ACC, ACC glutamate levels are lower-than-normal and lower glutamate levels are associated with more severe clinical symptoms and lower cognitive function in other clinical stages of schizophrenia spectrum disorders including non-treatment-resistant schizophrenia and FEP. It is important to consider how these findings are associated with the NMDA receptor hypofunction hypothesis. A recent MRS study reported no acute effects of ketamine administration on ACC Glx levels in healthy subjects [39]. Some previous studies suggested elevated glutamate levels following NMDA receptor hypofunction (e.g., ketamine-evoked glutamate elevation in mice hippocampus) [40]. However, to the best of our knowledge, there has been no direct evidence of increased ACC glutamate levels induced by NMDA receptor hypofunction. As mentioned above, a pre-existing decrease in synaptic glutamate levels could theoretically result in NMDA receptor hypofunction. Thus, we assume that the lower-than-normal levels of ACC glutamate found in non-treatment-resistant schizophrenia and FEP should be in favor of the glutamate hypothesis. Therefore, it can be hypothesized that lower ACC Glx levels may be related to psychotic characteristics at an early stage of psychotic disorder.

The associations between longitudinal changes in psychotic symptoms and those in ACC glutamatergic function in patients with schizophrenia spectrum disorders are almost unknown so far. Some recent longitudinal MRS studies have explored changes in ACC glutamatergic levels over antipsychotic treatment periods [41–43], but they reached different conclusions. For example, Bojesen et al. reported a lower glutamate-to-creatine ratio in the dorsal ACC in antipsychotic-naïve patients with FEP both at baseline and after antipsychotic treatment [43]. In addition, some studies reported different glutamatergic levels over antipsychotic treatment periods between ACC and another region [43]. Kubota et al. conducted a meta-analysis and reported decreased frontal Glx levels after treatment in patients with schizophrenia [44]. The different results across studies may be because of differences in brain regions of interest, in antipsychotic compounds used at baseline and during treatment, and in stages of schizophrenia spectrum disorders. To the best of our knowledge, there is no medium- or large-scale longitudinal study of ACC glutamatergic function in subjects at high-risk for psychosis, despite there being a previous small-scale study [45]. In addition, there is no longitudinal study of the association between psychotic symptoms and ACC glutamatergic function in subjects who keep antipsychotic-naïve throughout the studied period.

Subclinical psychotic experiences, also called psychotic-like experiences, can be found in the general population as a subthreshold phenotype, implying an underlying continuum of psychosis from subclinical to clinical levels. Longitudinal birth cohort studies have revealed that subclinical psychotic experiences in early adolescence are a risk factor for the later onset of schizophrenia [46]. Fisher et al. reported that children with psychotic symptoms at age 11 were at an elevated risk of developing schizophrenia by age 38 (relative risk 7.24) when compared to those without [47]. In addition, according to Zammit et al., the risk of psychotic disorders even at age 18 was greater in those with suspected as well as definite psychotic experiences at age 12 [48], suggesting that subclinical psychotic experiences in early adolescence can be representative to psychotic experiences around the time of the first episode. Furthermore, a recent study on data from Adolescent Brain Cognitive Development℠ study reported that early adolescents with persistent distressing subclinical psychotic experiences were likely to show large psychopathological and cognitive impairment [49]. Although a previous meta-analysis showed a 5% prevalence rate of subclinical psychotic experiences in the general population [50], the rate of subclinical psychotic experiences may be much higher among early adolescents. According to Kelleher et al., psychotic symptoms were reported by 21% of the early adolescents (ages 11–13 years) and 7% of the mid-adolescents (ages 13–16 years) [51]. Thus, it is rational to focus on subclinical psychotic experiences in a general early adolescent population. However, as far as we know, no previous study has explored the association between ACC glutamatergic levels and subclinical psychotic experiences in early adolescents.

Early life stress is a risk factor for schizophrenia onset. Increased emotional and social stress is associated with psychosis and subclinical psychotic experiences [52]. Bullying is a major social problem affecting adolescents all over the world. Being bullied in adolescence is associated with adverse mental health outcomes including psychotic symptoms [53, 54]. On the other hand, help-seeking behavior of adolescent bullied victims has a positive effect on their outcomes, which means that help-seeking is a protective coping strategy against stressors such as being bullied [55, 56]. However, it remains unclear how environmental emotional/social stressors biologically cause subclinical psychotic experiences in adolescents. Previous studies reported associations between stress-related factors and ACC glutamatergic function. Ho et al. reported a positive correlation in depressed adolescents between dorsal ACC glutamate levels and levels of pro-inflammatory cytokines, that may be induced by psychological stress [57]. Naismith et al. revealed a positive correlation in young adults with affective disorders between ventral ACC Glx levels and delayed circadian phase, which may be related to psychological distress [58]. Other previous studies reported associations between environmental emotional/social stressors and ACC glutamatergic function. Ventral ACC glutamatergic levels are lower-than-normal in youths traumatized by a natural disaster [59] and a terror attack [60], which are rare but not common environmental stressors. To the best of our knowledge, no previous study has explored the effects of commonly experienced environmental emotional/social stressors on ACC glutamatergic function in human adolescents. In addition, no previous study has explored its moderating effects of a help-seeking strategy, while the effect of a help-seeking strategy may be different with and without stressors.

Gamma-aminobutyric acid (GABA) is synthesized from glutamate by glutamate decarboxylase (GAD). GABA functions as an inhibitory neurotransmitter, while glutamate acts as an excitatory neurotransmitter. GABAergic neurons, including parvalbumin-expressing interneurons, orchestrate gamma-band oscillation [61], contributing to higher cognitive function. Expression of GAD67 messenger ribonucleic acid and other GABA-related transcripts is decreased in the postmortem ACC of patients with schizophrenia [62–64]. Recent MRS meta-analytic studies indicate lower-than-normal GABA levels in the dorsal ACC but not the ventral ACC in schizophrenia [65] and FEP [28]. Additionally, GABA levels are reported as normal in the ventral ACC in individuals at ultra-high risk for psychosis [65]. However, as far as we know, no previous study has investigated the association between ACC GABA levels and subclinical psychotic experiences in early adolescents. Previous MRS studies explored associations of psychological stressors with ACC GABA levels in adults, but the results were inconclusive [66–69]. To the best of our knowledge, there is a lack of exploration into the effects of commonly experienced environmental emotional/social stressors on ACC GABAergic function in early adolescents.

In this context, we hypothesized an association over time between ACC glutamatergic levels and subclinical psychotic experiences in the general adolescent population. Specifically, it was hypothesized that the association of these two variables should be consistent over time and that the association between differences over time in these two variables should be present. In addition, we hypothesized an effect of bullying victimization on ACC glutamatergic levels and its moderating effect of help-seeking intentions in the general adolescent population. We thought that the ventral (pregenual) ACC was a better region to focus on compared to the dorsal ACC to test our hypotheses. This was because both being bullied and having psychotic experiences, especially the former, are related to emotional dysregulation [22, 70], and because several previous neuroimaging studies reported the roles of the ventral ACC both in psychosis [71–73] and in emotional social stress [74–76]. Thus, in this study, we explored the association of pregenual ACC glutamate levels with subclinical psychotic experiences over time using a two-time-point longitudinal dataset with more than 200 adolescents recruited from the Tokyo TEEN Cohort (TTC) study [77, 78]. We measured pregenual ACC Glx levels using MRS technique. Additionally, we investigated the association between emotionally and socially stressful events and glutamatergic function. Specifically, we explored the effects of bullying victimization as well as of help-seeking intentions on pregenual ACC Glx levels. For a contrast analysis, GABA levels were also analyzed under our hypothesis that there is no association of GABAergic function in the pregenual ACC with subclinical psychotic experiences or bullying victimization/help-seeking intentions in the early adolescents. We expected this study to elucidate neural substrates of adolescent psychotic experiences according to the glutamate hypothesis of schizophrenia.

## Materials and methods

### Overview and recruitment

This study was conducted as part of the population-neuroscience study of the TTC (pn-TTC) study, in which early adolescents were recruited from the general population [78]. The participants in the pn-TTC study were subsampled from a larger participant group in the TTC study, a large-scale longitudinal population-based cohort survey involved 3,171 early adolescents living in the Tokyo metropolitan area [77]. Written informed assent and consent were obtained from each participant and her/his primary parent, respectively, before participation. All protocols were approved by the research ethics committees of the Graduate School of Medicine and Faculty of Medicine at the University of Tokyo (approval nos. 3150, 10057, and 10069), Tokyo Metropolitan Institute of Medical Science (approval no. 12–35), and the Graduate University for Advanced Studies (SOKENDAI) (approval no. 2012002). The details of the overview and recruitment are described in Supplementary Method 1 and elsewhere [20, 21]. Age and sex of each participant were identified by self-report. In addition, trends in gender dysphoria were identified by self-report, although they were not analyzed in this study. Race or ethnicity was not asked in the TTC study, because most participants in the TTC study had the same ethnic and racial origin. The pn-TTC data were collected from October 2013 to February 2016 for Time 1 and from April 2016 to March 2018 for Time 2. The interval between the two visits in the pn-TTC study was set to approximately two years. MRS data were obtained from 253 adolescents (134 boys and 119 girls, mean 11.5 y) at Time 1 and 237 adolescents (128 boys and 109 girls, mean 13.6 y) at Time 2. We had previously published studies including MRS data also used in this study [20, 21]. Data were analyzed from January 2019 to September 2023.

### Image acquisition and analysis

MRI scanning was conducted on a Philips Achieva 3T system (Philips Medical Systems, Best, The Netherlands) with an 8-channel receive head coil. Most participants visited twice, at Times 1 and 2, with an interval of approximately two years. Each participant underwent a series of MRI scans including fluid-attenuated inversion recovery (FLAIR), a T1 three-dimensional (3D) magnetization-prepared rapid gradient echo sequence (3D-MPRAGE), MR angiography (MRA), and MRS sequences. However, not all participants underwent all the above-mentioned sequences for various reasons, such as interruption of scanning due to participant fatigue. Similar to our previous studies, subjects with any abnormal brain organic findings detected by radiologists using 3D-MPRAGE, FLAIR, and MRA data were excluded from the current study.

**Fig. 1:**
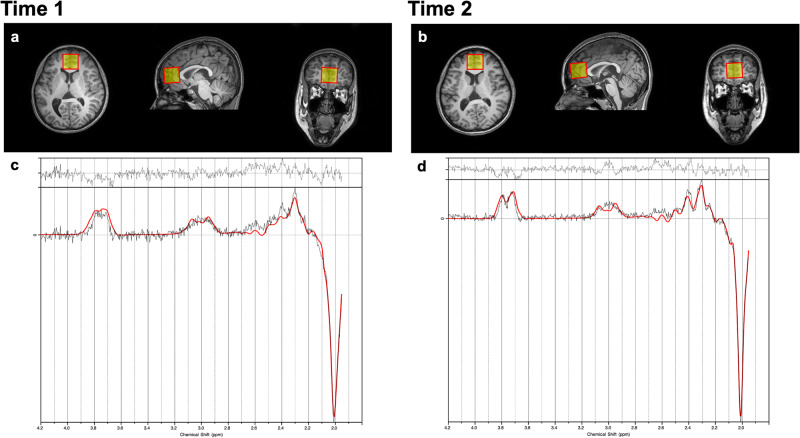
Longitudinal proton magnetic resonance spectroscopy (MRS) measurements of a representative participant. Voxel-of-interest (VOI) placements for the participant at Time 1 (**a**) and Time 2 (**b**) are demonstrated. A VOI for ^1^H MRS was placed at the anterior cingulate cortex (ACC) in 3D-T1 anatomical images (left, axial plane; middle, sagittal plane; right, coronal plane). VOIs were highlighted for visual purposes. The analysis results of ^1^H MRS spectra of the participant collected at Time 1 (**c**) and Time 2 (**d**) are shown. MEGA-editing was achieved with 15-ms Gaussian editing pulses applied at 1.90 ppm (ON) and 7.46 ppm (OFF) in alternate spectral lines. Actual spectra (the difference between edit ON and OFF spectra) (gray line) and their LCModel fits (red line) are displayed, where co-edited Glx signal peaks (~3.75 ppm) are clearly detectable.

For MRS, 1H-MRS spectra were collected from a 30 × 30 × 30 mm^3^ voxel of interest (VOI) in the pregenual ACC with the MEGA-PRESS method [79]. The reason the MEGA-PRESS method was chosen is that, while glutamate was the primary target metabolite in this study, it was deemed meaningful to explore not only glutamate but also GABA. The pregenual ACC VOI was positioned anterior and close to the corpus callosum genu tip and centered on the interhemispheric fissure (Fig. 1a, b). Spectral data were obtained using the following parameters: repetition time/echo time, 2000 ms/68 ms; 320 acquired transients; 2048 sample points; bandwidth, 2000 Hz; and 16-step phase cycle. Acquisition time was approximately 10 min 42 s. MEGA-editing was achieved with 15-ms Gaussian editing pulses applied at 1.90 ppm (ON) and 7.46 ppm (OFF) in alternate spectral lines. Water suppression was achieved using the multiply optimized insensitive suppression train (MOIST) water suppression techniques (for Philips scanners).

All MRS data were at first processed using the Gannet 3.2 toolbox [80]. MRS VOIs were coregistered to the corresponding T1-weighted images using GannetCoRegister. Regarding the longitudinal analysis, displacement over time of the center of the MRS VOI was calculated based on the coordinate of the MRS VOI’s center originating at the center of the anterior commissure after the coregistration of T1-weighted images to the MNI-ICBM152 template. In addition, using GannetSegment, T1-weighted images were segmented by Statistical Parametric Mapping 12 (SPM12, www.fil.ion.ucl.ac.uk/spm), and then the tissue fraction (*f*) of gray matter (GM), white matter (WM) and cerebrospinal fluid (CSF) for the VOI was quantified (Supplementary Fig. 1). Regarding the longitudinal analysis, the changes in the tissue fraction of GM, WM, and CSF for the VOI were calculated. Visual inspection was performed for coregistration and segmentation (Supplementary Fig. 2).

Next, MEGA-PRESS difference spectra were analyzed to obtain Glx levels with the LCModel package version 6.3 [81], a frequency domain spectral fitting program (Fig. 1c, d). A recent study reported that Glx levels estimated from MEGA-PRESS difference spectra explained 96 percent of the variance of the known Glx concentration of phantoms [82]. This fitting method provides the concentrations [institutional units (i.u.)] of the metabolites including Glx. The control parameter sptype for the LCModel analysis was set to mega-press-3. The LCModel software automatically performs frequency-and-phase correction based on the water-suppressed and unsuppressed reference spectra and automatically conducts water scaling and fitting to calculate neurometabolite levels by referencing to the unsuppressed water peak. For partial volume correction, the 1H-MRS visible water concentration (mM) in the VOI was calculated according to the formula [(43300*f*_GM_ + 35880*f*_WM_ + 55556*f*_CSF_)/(1 − *f*_CSF_)] provided by the LCModel manual (http://s-provencher.com/pub/LCModel/manual/manual.pdf), and subsequently inputted into the LCModel analysis. The concentrations of water (mM) in GM, WM, and CSF are 43300, 35880, and 55556, respectively. Cramer-Rao lower bounds (CRLB) were used to express uncertainties in quantifying metabolite levels. Only metabolite spectra with an LCModel-estimated uncertainty of <20% standard deviations (SDs) were included in this study to reject low-quality spectra.

Moreover, for a contrast analysis, all MRS data analyzed in the Glx analysis were processed for analysis of GABA plus macromolecule (GABA+) using GABA analysis toolkit ‘Gannet’ (Supplementary Fig. 3). Using GannetFit, edited GABA signals were fitted and GABA+ levels were assessed. GannetCoRegister and GannetSegment were used as described above. To correct for partial volume effects, data were processed using GannetQuantify, which combines modeled peak areas from GannetFit and voxel tissue fractions from GannetSegment with preset values for GABA and water relaxation and visibility [83]. Only metabolite spectra with Gannet fit error of <20% SDs were included in this study to reject low-quality spectra.

### Psychological and environmental evaluation

Data of subclinical psychotic experiences were obtained longitudinally at Times 1 and 2. Four questions administered in the Diagnostic Interview Schedule for Children (DISC-C) were used in the current study to assess subclinical psychotic experiences [46]. A total score of subclinical psychotic experiences can range from 0 (low) to 8 (high). Bullying victimization data were obtained at Time 1 using two self-administered questions and one parent-administered question [77, 84]. Participants were dichotomized into non-bullied victims (0) and bullied victims (1). Help-seeking intention data were obtained at Time 1. Help-seeking intentions were assessed using the same method as in our previous study [85]. Participants were dichotomized into those without help-seeking intentions (0) and those with help-seeking intentions (1). Information on socioeconomic status (SES) was obtained longitudinally (Times 1 and 2). SES was classified based on the annual household income and SES scores can range from 1 (low income) to 11 (high income) [77]. In principle, the averaged SES scores were included in the analysis models. In the present analysis, two subtests (information, picture completion) of the Wechsler Intelligence Scale for Children – Third Edition (WISC-III) were collected and used for calculation of intelligence quotient (IQ) at Time 1. Our rationale for only using the two subtests of WISC-III is as follows. The full version of the WISC-III was conducted for 28 children one year after the initial survey using the two subtests. Using multiple regression analysis with full IQ as a dependent variable and the results of the two subtests as independent variables, a formula for estimating IQ from the two subtests was created and the estimated IQ explained 78% of the variance of the full IQ [77]. Detailed explanations regarding psychological and environmental evaluation are described in the Supplementary Method 2.

### Statistical analysis

Power analyses were conducted (Supplementary Method 3). All statistical analyses, except where noted, were performed using Statistical Package for Social Science (SPSS) software version 27 (IBM Corp.). The statistical significance level was set to *p* < 0.05 (two-tailed). In principle, subjects with both Glx and subclinical psychotic experience data were included in our current analyses. Mean imputation was performed for missing covariates data.

First, we investigated the association of Glx levels with subclinical psychotic experiences at each time point. Partial Spearman’s correlation between Glx levels and subclinical psychotic experiences was assessed, adjusted for age at MRI scanning, sex, SES, and IQ. In addition, we investigated whether the Glx level changes and changes in subclinical psychotic experiences between the two time points were associated. Partial Spearman’s correlation between Glx level changes and changes in subclinical psychotic experiences between the two time points was assessed, adjusted for MRI scan interval, sex, SES, and IQ. Multiple testing correction with the Benjamini and Hochberg false discovery rate (FDR) method was performed using p.adjust in R 4.0.5.

Second, the effects of bullying victimization and help-seeking intention on Glx levels at Times 1 and 2 were investigated. Glx levels were adjusted for age, sex, SES, and IQ in a linear regression model. After checking whether all the assumptions were met, a three-factor mixed-design analysis of variance (ANOVA) was conducted with age-sex-SES-IQ-adjusted Glx levels as the dependent factor, bullying victimization and help-seeking intention as between-subjects factors, and time as a within-subjects factor.

Finally, we performed path analysis to determine the relationships among variables including bullying victimization, help-seeking intention, age-sex-SES-IQ-adjusted Glx levels, and subclinical psychotic experiences using partial least squares structural equation modeling (PLS-SEM), which is a non-parametric analysis technique. All subjects enrolled in the ANOVA study were included. Basically, in this analysis, paths from bullying victimization to Glx levels and paths from Glx levels and subclinical psychotic experiences were regarded as the main connections, and the moderating effects of help-seeking intention on paths from bullying victimization to Glx levels were also investigated. We created a time lagged model, where latent variables at two time points were separately included, and a latent change score model, where latent baseline (intercept) variables and latent change (slope) variables were included. The PLS-SEM analyses were implemented using the SmartPLS 4.0 software, which allows us to estimate direct and moderating (interaction) effects. Bootstrapping method with 5,000 random resamples was used. The fit of the PLS-SEM model was evaluated using standardized root mean residual (SRMR), which is provided by the SmartPLS 4.0 software. An SRMR value of less than 0.10 indicates an acceptable fit to the model [86]. Indirect effects were also assessed using PLE-SEM models.

For a contrast analysis, GABA+ levels were statistically analyzed in ways similar to those used in Glx analysis. The details are described in the Supplementary Method 4.

## Results

### Overview of the analyzed data

The flow chart of subject inclusion and exclusion is shown in Supplementary Table 1. Demographics and basic results in this study are presented in Table 1. For the longitudinal dataset analyzed (*n* = 157), the mean displacement of the center of the MRS VOI was 5.2 mm, equal to the diagonal of a 1.7 mm cube, and the mean changes in the tissue fraction of GM, WM, and CSF for the VOI were −2.7%, 1.7%, and 1.0%, respectively. Bullying victimization was not significantly associated with help-seeking intention (Supplementary Table 2).

**Table 1:** Demographics and basic results for subjects with both subclinical psychotic experience and Glx data.

	Time 1 (*N* = 219)		Time 2 (*N* = 211)		Times 1 and 2 (*N* = 157)	
	*N*/Mean	%/SD	*N*/Mean	%/SD	*N*/Mean	%/SD
Age (y)^a^	11.5	0.7	13.6	0.6	2.0^b^	0.5^b^
Sex						
Boys	114	52.1	111	52.6	85	54.1
Girls	105	47.9	100	47.4	72	45.9
SES^a^	8.1^c^	2.5^c^	8.3	2.5	8.2	2.5
IQ^a^	108.5	13.0	108.9	12.6	109.2	12.9
BV						
Negative	154	70.3	152	72.0	115	73.2
Positive	64	29.2	59	28.0	42	26.8
Missing	1	0.5	0	0.0	0	0.0
HSI						
Negative	41	18.7	36	17.1	27	17.2
Positive	176	80.4	172	81.5	129	82.2
Missing	2	0.9	3	1.4	1	0.6
SPEs ^a^	1.4	1.4	0.7	1.1	−0.7^b^	1.5^b^
Glx levels (i.u.)^a^	6.9	0.6	6.5	0.7	−0.4^b^	0.8^b^

*SD* standard deviation, *SES* socioeconomic status, *IQ* intelligence quotient, *BV* bullying victimization, *HSI* help-seeking intention, *SPE* subclinical psychotic experience, *Glx* combined glutamate-glutamine, *i.u.* institutional units.

^a^Means (standard deviations) are presented.

^b^Changes over time (Time 2 − Time 1) are presented.

^c^One subject had missing data.

### Associations over time between Glx levels and subclinical psychotic experiences

**Fig. 2:**
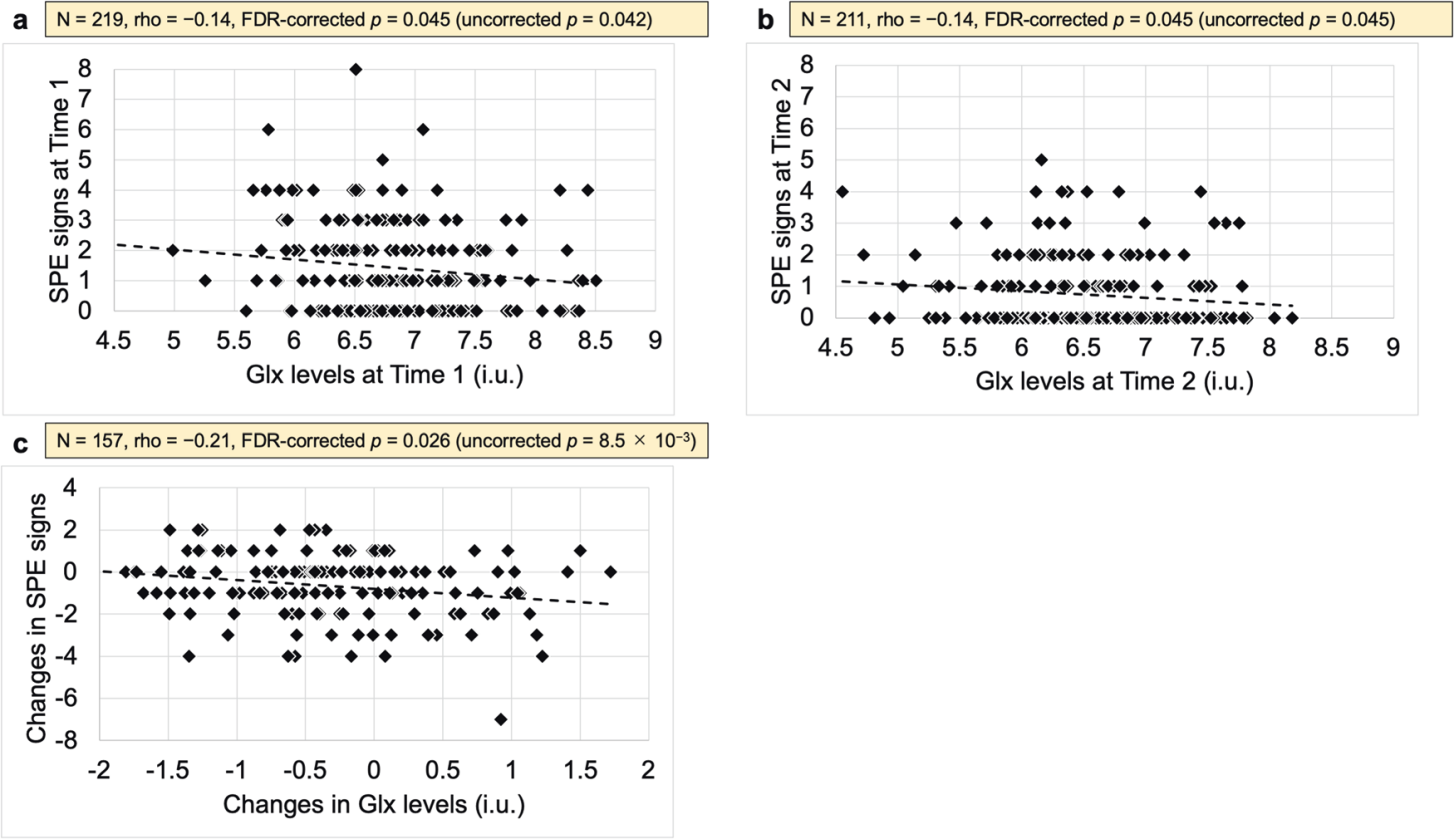
Associations over time between Glx levels and signs of subclinical psychotic experiences. **a** The association at Time 1, **b** the association at Time 2, and **c** the association between longitudinal changes are illustrated. Significant negative associations between Glx levels in the pregenual ACC and subclinical psychotic experiences at both Times 1 (**a**) and 2 (**b**) as well as between over-time changes in the two variables were found (**c**). Glx combined glutamate-glutamine, SPE subclinical psychotic experience, i.u. institutional units.

Associations over time between Glx levels and subclinical psychotic experiences were investigated. Subclinical psychotic experiences were significantly negatively associated with Glx levels at both time points (Time 1, *n* = 219, *ρ* = −0.14, FDR-corrected *p* = 0.045, uncorrected *p* = 0.042; Time 2, *n* = 211, *ρ* = −0.14, FDR-corrected *p* = 0.045, uncorrected *p* = 0.045) (Fig. 2a, b), and changes in subclinical psychotic experience over time were significantly negatively associated with Glx level changes (*n* = 157, *ρ* = −0.21, FDR-corrected *p* = 0.026, uncorrected *p* = 8.5 × 10^−3^) (Fig. 2c).

### Effects of bullying victimization and help-seeking intention on Glx levels

**Fig. 3:**
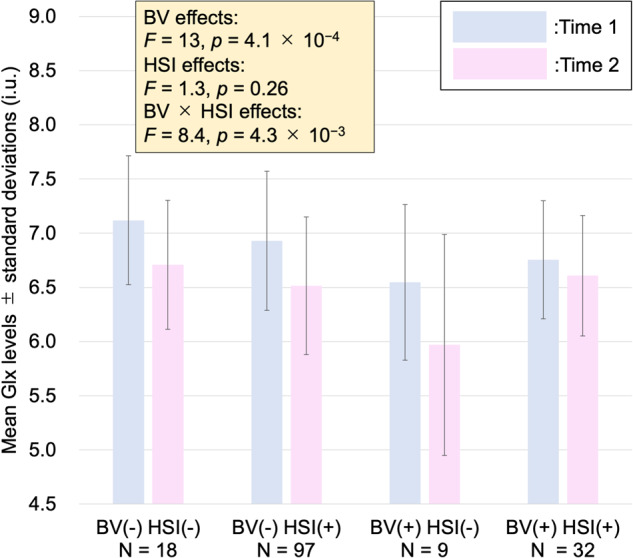
Effects of bullying victimization and help-seeking intention on longitudinal Glx levels. Light blue-colored bars represent Glx levels at Time 1, and pink-colored bars represent Glx levels at Time 2. Bullying victimization and bullying victimization × help-seeking intention interaction had significant effects on Glx levels. The bullied group showed lower ACC Glx levels than the non-bullied group for all participants. Post hoc tests revealed that while the help-seeking and non-help-seeking groups showed similar Glx levels within the non-bullied group, the help-seeking group showed higher Glx levels than the non-help-seeking group within the bullied group. BV bullying victimization, HSI help-seeking intention, Glx combined glutamate-glutamine, i.u. institutional units.

The effects of bullying victimization and help-seeking intention on Glx levels were investigated. All the assumptions of an ANOVA, such as the normal distribution of age-sex-SES-IQ-adjusted Glx levels, were met. The main effects of bullying victimization (*F* = 13, *p* = 4.1 × 10^−4^) and bullying victimization × help-seeking intention interaction effects (*F* = 8.4, *p* = 4.3 × 10^−3^) on age-sex-SES-IQ-adjusted Glx levels were significant (*n* = 156) (Fig. 3), while there were no significant effects of help-seeking intention (*F* = 1.3, *p* = 0.26), time (*F* = 0.31, *p* = 0.58), time × bullying victimization interaction (*F* = 0.038, *p* = 0.85), time × help-seeking intention interaction (*F* = 2.1, *p* = 0.15), or time × bullying victimization × help-seeking intention interaction (*F* = 1.7, *p* = 0.19). Specifically, the bullied group showed lower ACC Glx levels than the non-bullied group for all participants. Post hoc Bonferroni tests revealed that while the help-seeking and non-help-seeking groups showed similar Glx levels within the non-bullied group (*F* = 2.5, *p* = 0.12), the help-seeking group showed higher Glx levels than the non-help-seeking group within the bullied group (*F* = 6.0, *p* = 0.016).

### PLS-SEM analysis

**Fig. 4:**
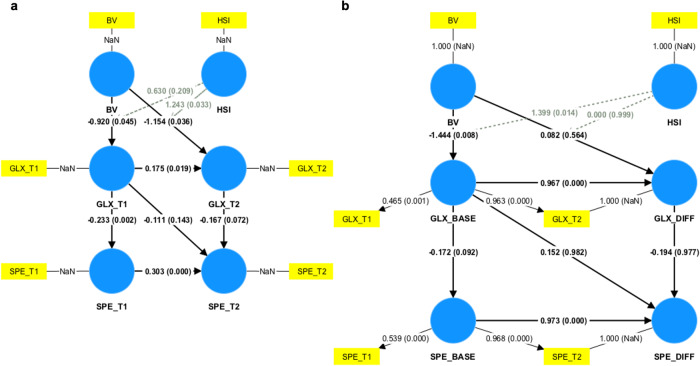
Results of PLS-SEM analysis for the associations among bullying victimization, help-seeking intention, Glx, and subclinical psychotic experiences. Path analyses were performed to determine the relationships among variables including bullying victimization, help-seeking intention, age-sex-SES-IQ-adjusted Glx levels, and subclinical psychotic experiences using PLS-SEM. Results of **a** the time lagged model and of **b** the latent change score model are illustrated here. Squares represent observed variables and circles represent latent variables. Solid arrows represent direct effects and dashed arrows represent moderation effects. Path coefficients (*p* values in parentheses) are shown on corresponding arrows. Indirect effects were also assessed. In the time lagged model (**a**), Glx levels at Time 1 had a significant total indirect effect on subclinical psychotic experience at Time 2, and bullying victimization had a trend-level significant total indirect impact on subclinical psychotic experience at Time 2. In the latent change score model (**b**), bullying victimization and bullying victimization × help-seeking intention interaction had significant total indirect effects on differences in Glx levels. BV bullying victimization, HSI help-seeking intention, GLX combined glutamate-glutamine, SPE subclinical psychotic experience, T1 Time 1, T2 Time 2 BASE baseline, DIFF difference.

Path analyses were performed to determine the relationships among variables including bullying victimization, help-seeking intention, age-sex-SES-IQ-adjusted Glx levels, and subclinical psychotic experiences using PLS-SEM. First, a time lagged model was created, and the PLS-SEM test revealed that the current model fitted the data well (n = 156, SRMR = 0.000) (Fig. 4a). Specifically, the path coefficient (PC) from bullying victimization to Glx levels at Time 1 (PC = −0.92, *p* = 0.045), PC from bullying victimization to Glx levels at Time 2 (PC = −1.16, *p* = 0.036), PC from Glx levels at Time 1 to Glx levels at Time 2 (PC = 0.18, *p* = 0.019), PC from Glx levels at Time 1 to subclinical psychotic experiences at Time 1 (PC = −0.23, *p* = 0.002), and PC from subclinical psychotic experiences at Time 1 to subclinical psychotic experiences at Time 2 (PC = 0.303, *p* = 0.000) were significant, whereas the PC from Glx levels at Time 2 to subclinical psychotic experiences at Time 2 was trend-level significant (PC = −0.17, *p* = 0.072). The PC from Glx levels at Time 1 to subclinical psychotic experiences at Time 2 was not significant (PC = −0.11, *p* = 0.14). In addition, help-seeking intention significantly moderated the path from bullying victimization to Glx levels at Time 2 (PC = 1.2, *p* = 0.033) but not the path from bullying victimization to Glx levels at Time 1 (PC = 0.63, *p* = 0.21). As for total indirect effects, Glx levels at Time 1 had a significant impact on subclinical psychotic experiences at Time 2 (PC = −0.10, *p* = 0.005), and bullying victimization had a trend-level significant impact on subclinical psychotic experiences at Time 2 (PC = 0.39, *p* = 0.057). As for specific indirect effects, subclinical psychotic experiences at Time 1 significantly mediated the association between Glx levels at Time 1 and subclinical psychotic experiences at Time 2 (PC = −0.071, *p* = 0.018). No further indirect effects were observed.

Next, a latent change score model was created, while the PLS-SEM test revealed that the current model fitted the data slightly poorly (*n* = 156, SRMR = 0.11) (Fig. 4b). Specifically, the PC from bullying victimization to baseline Glx levels (PC = −1.4, *p* = 0.008), PC from baseline Glx levels to differences in Glx levels (PC = 0.97, *p* = 0.000), and PC from baseline subclinical psychotic experiences to differences in subclinical psychotic experiences (PC = 0.97, *p* = 0.000) were significant, while the PC from baseline Glx levels to baseline subclinical psychotic experiences (PC = −0.17, *p* = 0.092) was trend-level significant. The PC from bullying victimization to differences in Glx levels (PC = 0.082, *p* = 0.56), PC from baseline Glx levels to differences in subclinical psychotic experiences (PC = 0.15, *p* = 0.98), and PC from differences in Glx levels to differences in subclinical psychotic experiences were non-significant (PC = −0.19, *p* = 0.98). In addition, help-seeking intention significantly moderated the path from bullying victimization to baseline Glx levels (PC = 1.4, *p* = 0.014) but not the path from bullying victimization to differences in Glx levels (PC = 0.000, *p* = 1.0). As for total indirect effects, bullying victimization and bullying victimization × help-seeking intention interaction had significant effects on differences in Glx levels (bullying victimization, PC = −1.4, *p* = 0.007; bullying victimization × help-seeking intention, PC = 1.4, *p* = 0.012). As for specific indirect effects, baseline Glx levels significantly mediated the association between bullying victimization and differences in Glx levels (PC = −1.4, *p* = 0.007) and the association between bullying victimization × help-seeking intention interaction and differences in Glx levels (PC = 1.4, *p* = 0.012). Baseline subclinical psychotic experiences mediated the association, at a trend level, between baseline Glx levels and differences in subclinical psychotic experiences (PC = −0.17, *p* = 0.090). No further indirect effects were observed.

### Contrast analysis

Analyses of GABA+ levels did not reveal any significant results (Supplementary Results 1, Supplementary Figs. 4–6).

## Discussion

In this study, we revealed a negative association between Glx levels in the pregenual ACC and subclinical psychotic experiences at both Times 1 and 2, as well as for changes over time. Moreover, we found significant bullying victimization and bullying victimization × help-seeking intention interaction effects on Glx levels. Specifically, bullying victimization decreased Glx levels, whereas help-seeking intentions increased Glx levels only in adolescents with bullying victimization. Finally, our PLS-SEM analysis revealed associations among bullying victimization, help-seeking intentions, Glx levels, and subclinical psychotic experiences.

We revealed a negative association between Glx levels in the pregenual ACC and subclinical psychotic experiences at both Times 1 and 2 (Fig. 2a, b). To the best of our knowledge, this is the first longitudinal study of the association between glutamatergic function in the pregenual ACC and subclinical psychotic experiences in the general adolescent population, and the first to reveal a negative association between Glx levels and subclinical psychotic experiences at two time points. Recent 7-tesla MRS studies have reported lower glutamate and glutamine levels in the dorsal ACC in patients with schizophrenia [87] and FEP [32, 33, 88] compared to healthy controls. This seems in line with our findings despite different subregions of interest within the ACC and different subclinical/clinical stages. In addition, other recent 7-tesla MRS studies have reported lower glutamate levels in the pregenual ACC in patients with residual schizophrenia [89] and FEP [90] compared to healthy subjects. This seems in line with our findings despite different subclinical/clinical stages, and it is suggested that our findings may extend the already known findings of lower glutamate levels in the pregenual ACC after psychosis onset to those before onset (subclinical psychotic experiences). In contrast, as described above, recent meta-analyses reported higher ACC glutamate levels [91] and higher Glx and glutamate levels in the dorsal ACC (middle cingulate cortex) in treatment-resistant schizophrenia [28]. Combining our findings with the above studies, it is suggested that ACC glutamate substrates in adolescent subclinical psychotic experiences, FEP, and residual schizophrenia may be different from those in treatment-resistant schizophrenia. As mentioned above, the results on ACC glutamatergic levels in genetic high-risk subjects are so far controversial [7, 35]. In addition, a recent 7-tesla MRS study has reported no alterations in glutamate levels in the dorsal ACC in patients with 22q11.2 deletion syndrome, characterized by an increased risk of psychosis [92]. Taken together, genetic risk factors for psychosis may have a smaller effect on glutamatergic levels in the ACC (especially its pregenual part) compared to environmental factors, examples of which will be discussed below. Future studies will be necessary to reveal in more detail how various risk factors for psychosis affect ACC glutamatergic levels.

We revealed a negative association of ACC Glx level changes with changes in subclinical psychotic experiences over a period of two years (Fig. 2c). This is concordant with our findings of a negative association between ACC Glx levels and subclinical psychotic experiences at each time point (Fig. 2a, b). Given negative associations between Glx levels and subclinical psychotic experiences at two time points as well as for over-time changes, our findings suggest that Glx levels in the pregenual ACC may in the future be able to help assess the current state of or within-person changes in psychotic experiences, although further studies are needed to verify its utility.

We found significant bullying victimization and bullying victimization × help-seeking intention interaction effects on Glx levels (Fig. 3). Specifically, the bullied group showed lower ACC Glx levels than the non-bullied group for all participants. To the best of our knowledge, this is the first study to elucidate the association of commonly experienced environmental emotional/social stressors with glutamatergic function in human adolescents. Previous studies have reported lower ACC glutamatergic levels in traumatized youths [59, 60]. Recent mouse studies have reported that exposure to social stress decreases Glx levels in the medial prefrontal cortex (mPFC) [93] and reduces the ratio of vesicular glutamate 1 transporter to vesicular GABA transporter, representing the ratio of glutamate to GABA in the synapse, in the prelimbic cortex of the mPFC [94], homologous to the human pregenual ACC [95]. These studies are in line with our findings. A recent adolescent arterial spin-labeling study has reported increased activation in the ventral ACC during a social exclusion task, which was positively correlated with the extent of previous exposure to bullying victimization [74]. We assume that Glx levels in the ventral ACC may affect the regional blood flow changes in response to social rejection. In summary, our finding regarding the negative effect of bullying victimization on ACC Glx levels suggests a neurobiological basis of the psychosis-inducing effects of bullying victimization. In addition, while the help-seeking and non-help-seeking groups showed similar Glx levels within the non-bullied group, the help-seeking group showed higher Glx levels than the non-help-seeking group within the bullied group. A recent study has reported increased glutamate and Glx levels in the ventral ACC after brief mindfulness training, which is a preventive strategy for psychological well-being [96]. It is suggested that acts of self-care such as help-seeking intentions and meditation may promote psychological well-being through increased Glx levels in the ventral ACC. Our finding suggests a neurobiological substrate for the antipsychotic role of help-seeking intentions against adversity. Combining with our other results discussed above (Fig. 2a–c), our findings elucidated neural substrates underlying the association among bullying victimization, help-seeking intention, and subclinical psychotic experiences according to the glutamate hypothesis of schizophrenia. In addition, it should be crucial for supporting healthy adolescent brain development to implement adolescent education to prevent bullying and promote help-seeking behavior in case of being bullied.

Our PLS-SEM analysis revealed associations among bullying victimization, help-seeking intentions, Glx levels, and subclinical psychotic experiences. Specifically, with a time lagged model (Fig. 4a), Glx levels at Time 1 had a significant indirect total impact on subclinical psychotic experiences at Time 2, which suggests that Glx levels affect subclinical psychotic experiences even two years later. In addition, subclinical psychotic experiences at Time 1 significantly mediated the association between Glx levels at Time 1 and subclinical psychotic experiences at Time 2, which suggests that lower Glx levels may result in persistent subclinical psychotic experiences. Given that persistent subclinical psychotic experiences are associated with later transition to clinical psychosis [97] and poor psychological outcomes in adolescence [98], it is suggested that lower Glx levels in early adolescence may act as a risk factor for various psychological symptoms through persistent subclinical psychotic experiences. Moreover, bullying victimization had a trend-level significant indirect effect on subclinical psychotic experiences at Time 2. To the best of our knowledge, this is the first study to reveal the neurometabolic basis of the phenomenon on the association between bullying victimization and subsequent psychotic symptoms [53, 54].

In addition, with a latent change score model (Fig. 4b), the PC from bullying victimization to baseline Glx levels was significant and the PC from baseline Glx levels to baseline subclinical psychotic experiences was trend-level significant. This finding is mostly in line with our above-mentioned results (Figs. 2a,b, 3, 4a). However, the PC from differences in Glx levels to differences in subclinical psychotic experiences was not significant, suggesting no relationships between latent change scores of Glx levels and of subclinical psychotic experiences. This finding is not in line with our above-mentioned results on the association of ACC Glx level changes with changes in subclinical psychotic experiences over a period of two years (Fig. 2c), which may have to be cautiously interpreted. Moreover, help-seeking intention significantly moderated the path from bullying victimization to baseline Glx levels. Bullying victimization and bullying victimization × help-seeking intention interaction had significant effects on differences in Glx levels. This finding is in line with our above-mentioned results (Figs. 3 and 4a).

Our contrast analysis revealed no association of pregenual ACC GABA+ levels with subclinical psychotic experiences (Supplementary Fig. 4), which is in concordance with our hypothesis. Our findings are consistent with previous research showing no alterations in pregenual ACC GABA+ levels in patients with schizophrenia, those with FEP, and high-risk individuals [28, 65]. Rowland et al. reported lower-than-normal ACC GABA+ levels in older but not younger patients with schizophrenia and their steeper-than-normal age-related decline in schizophrenia [99]. Thus, it may be suggested that pregenual ACC GABA+ levels are likely to be normal in the early stages of schizophrenia spectrum disorders, although further studies are required to confirm this suggestion. An adolescent electroencephalogram study from our group reported a positive association between longitudinal changes in psychological difficulties and those in auditory duration mismatch negativity, a marker for decreased glutamatergic neurotransmission, while no such association was found in the gamma-band auditory steady-state response, reflecting GABAergic interneuron function [100]. It is suggested that this supports our current findings despite different modalities and brain regions of interest. We also found no association of pregenual ACC GABA+ levels with bullying victimization or help-seeking intentions (Supplementary Fig. 5). A recent study of healthy adults reported no association between ACC GABA levels and perceived stress [101], supporting our findings. Further studies are required to explore this phenomenon in more detail.

This study had several limitations. First, neurometabolite levels measured using MRS include not only synaptically acting neurotransmitters but also pools (not used as neurotransmitters). Thus, the exact synaptic glutamate signaling status could not be assessed using this technique. Second, accurate separate quantification of glutamate and glutamine, whose molecular structures are very similar, is usually challenging with MRS techniques, especially the MEGA-PRESS method, due to the low efficiency in editing signals of the two metabolites [82]. Thus, Glx has often been analyzed instead of each of the two metabolites. In studies that report findings on Glx levels including our current study, Glx levels are usually interpreted as reflecting glutamatergic function. Although this is at least partly correct since Glx is the combined pool of glutamate and glutamine and glutamate is synthesized from glutamine, it should be noted that Glx levels do not perfectly account for the glutamatergic function. Third, our results using Glx estimates from MEGA-PRESS difference spectra should be carefully interpreted as some previous studies reported a positive but relatively low correlation between Glx estimates from MEGA-PRESS difference spectra and Glx estimates from spectra with the widely used PRESS sequence [102]. In contrast, as mentioned above, MEGA-PRESS difference spectra can mostly accurately estimate Glx concentrations of phantoms [82]. Further studies are expected to explore to what extent or how accurately MEGA-PRESS difference spectra can estimate actual Glx levels in the human brain. Fourth, the acquisition of spectra was limited to only one VOI and only one MRS sequence in this study because of time constraints and our desire to avoid participants tolerating undue stress. It would have been ideal to acquire spectra from another VOI as the control region and to add another MRS sequence such as PRESS. Fifth, the sample size of the bullied and non-help-seeking group was small (*n* = 9). Thus, our results should be cautiously interpreted. Sixth, while our study and some previous studies revealed similar findings of lower Glx levels in subjects with higher psychotic features [32, 33, 88, 90], this similarity should be carefully interpreted. As mentioned above, psychotic experiences at the age of around 12 are a risk factor for psychotic disorders at age 18 [48]. However, it is unclear so far to what extent and how accurately psychotic experiences in early adolescence can be linked to being at clinical high-risk or the onset of FEP. Further studies are expected to solve this question. Finally, adolescent patients with schizophrenia were not examined in this study, as those with evident psychiatric or neurological disorder were excluded. However, all the participants were drug naïve for psychotropic drugs in this study, which should be rather a strong point for adolescent brain imaging research.

To the best of our knowledge, this is the first study to reveal a longitudinal trajectory of the association between ACC glutamatergic function and subclinical psychotic experiences in the general adolescent population and to elucidate the effect of commonly experienced environmental emotional/social stressors on glutamatergic function, which can be related to psychosis. Our findings may deepen the understanding of how environmental emotional/social stressors induce impaired glutamatergic neurotransmission that could be the underpinning of liability for psychotic experiences in early adolescence.

### Supplementary information

Supplementary Information

## Data Availability

The datasets generated during and/or analyzed during the current study are not publicly available due to ethical reasons but are available from the corresponding author on reasonable request.
