# Supplementary material for: Longitudinal trajectories of anterior cingulate glutamate and subclinical psychotic experiences in early adolescence: the impact of bullying victimization
Source: Mol Psychiatry. 2024 Jan 5;29(4):939–50. doi: 10.1038/s41380-023-02382-8 (PMC11176069; doi:10.1038/s41380-023-02382-8)
Supplement: Supplementary file 1 — Supplementary Information [file 41380_2023_2382_MOESM1_ESM.pdf]

Supplementary Information

Longitudinal trajectories of anterior cingulate glutamate and subclinical psychotic experiences in early adolescence: The impact of bullying victimization

Supplementary Method 1: Overview and recruitment

The detailed methods for participant recruitment were described in our previous study.[1, 2] The current study was performed as part of the population-neuroscience study of the Tokyo TEEN Cohort (TTC) project (pn-TTC), in which approximately 300 early adolescents participated and biological markers including magnetic resonance (MR) imaging (MRI), deoxyribonucleic acid (DNA) methylations, and stress and gonadal hormones were measured.[3] The participants in the pn-TTC study were subsampled from a larger participant group in the TTC study. The TTC study is a large-scale longitudinal population-based cohort survey in the Tokyo metropolitan area, in which 3,171 early adolescents have participated.[4] Among the participants in the TTC survey, those who showed interest in the pn-TTC study were regarded as candidate participants. The included participants were enrolled in the pn-TTC study approximately one year after participation in the TTC study. To check for any non-negligible sampling bias in the pn-TTC subsample, we compared the basic attributes acquired in the TTC study between the participants and the non-participants in the pn-TTC study. We found no significant differences in age, sex, socioeconomic status (SES), or intelligence quotient (IQ) ($p >$ 0.20). We confirmed that the pn-TTC subsample was representative of the original TTC study population.

Written informed assent was obtained from each participant and written informed consent was obtained from the participant's primary parent before participation. All protocols were approved by the research ethics committees of the Graduate School of Medicine and Faculty of Medicine at the University of Tokyo (approval nos. 3150, 10057, and 10069), Tokyo Metropolitan Institute of Medical Science (approval no. 12–35), and the Graduate University for Advanced Studies (SOKENDAI) (approval no. 2012002). All studies were performed in accordance with the relevant guidelines and regulations.

Prior to MRI scanning, participants were introduced to a mock scanner to acclimatize to the MRI scanner environment and to practice lying still during the scan. The exclusion criteria for participation included (a) evident psychiatric or neurological disorder (e.g., autism spectrum disorder, attention deficit hyperactivity disorder, Down syndrome, or epilepsy); (b) visual or auditory impairment (except myopia); (c) endocrinological disease, or disease that might affect the hypothalamic-pituitary-adrenal axis function (e.g., diabetes mellitus, thyroid disease, or renal dysfunction), gonadotropic dysfunction, or adrenal dysfunction; (d) recent or long-term use of drugs that might influence the central nervous system (e.g., steroid hormones and antihistamines); (e) history of head trauma with loss of consciousness for five minutes or more; (f) metal implants (except titanium) in the body; and (g) unrest during the MRI practice session.

Supplementary Method 2: Psychological and environmental evaluation

Subclinical psychotic experiences

Subclinical psychotic experience data were obtained longitudinally at Times 1 and 2. Four questions administered in the Diagnostic Interview Schedule for Children (DISC-C),[5] which were also used in later studies,[6] were used in the current study to assess subclinical psychotic experiences: (a) “Have other people ever read your mind?” (b) “Have you ever had messages sent just to you through television or radio?” (c) “Have you ever thought that people are following you or spying on you?”; and (d) “Have you heard voices other people can't hear?” Three responses of “Yes, definitely,” “Maybe,” and “No, never” are possible, and scored as 2 points, 1 point, and 0 points, respectively. The total score was calculated by adding up the scores for all questionnaires, and its possible range is from 0 to 8. Additionally, these questions have been used in previous neuroimaging studies.[7-9]

Bullying victimization

Bullying victimization data obtained at Time 1 were used in this study. Children were asked two questions: “How often have you been bullied at school in the past two months?” and “How often have you been bullied outside school in the past two months?” Similarly, primary parents were asked the following question: “How often has your child been bullied in the past two months?” The participants were asked to choose one of five responses: “never,” “once or twice in two months,” “twice or three times a month,” “once a week,” and “several times a week.”[4, 10] All answers other than “never” were interpreted as being bullied.[11] Because the reproducibility of the evaluation for bullying victimization is low between children and parents, similarly to our previous study, disclosure by either a child or a primary parent of being bullied resulted in a categorization as a bullied victim.[11] A dummy variable was defined for the bullying victimization variable, with 1 indicating a bullied victim and 0 indicating a non-bullied victim.

Help-seeking intention

Help-seeking intention data obtained at Time 1 were used in the current study. Help-seeking intentions were assessed using the same method as in our previous study,[12] which was similar to that used in a large Australian study intended to identify a child with depression.[13] We called the child Taro, which is one of the most popular boy names in Japan. The description is as follows: “For the last several weeks, Taro has been feeling unusually sad. He is tired all the time and has trouble sleeping at night. Taro doesn't feel like eating and has lost weight. He can't keep his mind on his studies, and his grades have dropped. He puts off making any decisions, and even day-to-day tasks, such as studying and extracurricular activities, seem too much for

him. His parents and teachers are very concerned about him." This vignette was written to satisfy the diagnostic criteria for major depression according to the Diagnostic and Statistical Manual of Mental Disorders, Fourth Edition (DSM-IV) and International Statistical Classification of Diseases and Related Health Problems: 10th revision (ICD-10). Children were asked whether they would seek help from others if they were in the same situation as the boy in the vignette. The response options were, "I would consult someone immediately" and "I would wait and see without consulting anyone." Here, we did not specify the source of help, because the focus of this study was the intention to seek help from anyone and not the source of help. A dummy variable was defined for the help-seeking intention variable, with 0 indicating being non-help-seeking and 1 indicating being help-seeking.

Socioeconomic status

In the present analysis, SES was classified based on the annual household income with the following ordinal variables:1) ≤ Japanese yen (JPY) 999,999; 2) JPY 1,000,000–JPY 1,999,999; 3) JPY 2,000,000–JPY 2,999,999; 4) JPY 3,000,000–JPY 3,999,999; 5) JPY 4,000,000–JPY 4,999,999; 6) JPY 5,000,000–JPY 5,999,999; 7) JPY 6,000,000–JPY 6,999,999; 8) JPY 7,000,000–JPY 7,999,999; 9) JPY 8,000,000–JPY 8,999,999; 10) JPY 9,000,000–JPY 9,999,999; and 11) JPY 10,000,000 (JPY 100 ≈ United States dollar [USD] 1 as of the data collection period).[4] SES data were obtained longitudinally at Times 1 and 2, and the average SES was included in the analysis models. In subjects for whom SES data were collected only at one time point, data from one time-point were included in the analysis models.

Intelligence quotient

In the present analysis, two subtests (information, picture completion) of the Wechsler Intelligence Scale for Children – Third Edition (WISC-III)[14] were collected and used for calculation of IQ at Time 1. Our rationale for only using the two subtests of WISC-III is as follows. The full version of the WISC-III was conducted for 28 children one year after the initial survey using the two subtests. Using multiple regression analysis with full IQ as a dependent variable and the results of the two subtests as independent variables, a formula for estimating IQ from the two subtests was created and the estimated IQ explained 78% of the variance of the full IQ.[4]

Supplementary Method 3: Power analysis

Using G*Power 3.1.9.6,[15] a priori statistical power analyses were performed to estimate the minimum sample size. The minimum sample size for correlational analysis was

estimated at 84 with effect size $\rho = 0.30$, alpha = 0.05 (two-tailed), and power = 0.8. The minimum sample size for repeated measures analysis of variance (ANOVA) was estimated at 98 with effect size $f = 0.25$, alpha = 0.05, power = 0.8, number of groups = 2, and number of measurements = 2.

Supplementary Method 4: Statistical analysis for GABA+ levels

First, we investigated the association of gamma-aminobutyric acid (GABA) plus macromolecule (GABA+) levels with subclinical psychotic experiences at each time point. Partial Spearman's correlation between GABA+ levels and subclinical psychotic experiences was assessed, adjusted for age at MRI scanning, sex, SES, and IQ. In addition, we investigated whether the GABA+ level changes and changes in subclinical psychotic experiences between the two time points were associated. Partial Spearman's correlation between GABA+ level changes and changes in subclinical psychotic experiences between the two time points was assessed, adjusted for MRI scan interval, sex, SES, and IQ. Multiple testing correction was performed using false discovery rate (FDR) analysis using p.adjust in R 4.0.5.

Second, the effects of bullying victimization and help-seeking intention on GABA+ levels at Times 1 and 2 were investigated. GABA+ levels were adjusted for age, sex, SES, and IQ in a linear regression model. After checking whether all the assumptions were met, a three-factor mixed-design ANOVA was conducted with age-sex-SES-IQ-adjusted GABA+ levels as the dependent factor, bullying victimization and help-seeking intention as between-subjects factors, and time as a within-subjects factor.

Finally, we performed path analysis to determine the relationships among variables including bullying victimization, help-seeking intention, age-sex-SES-IQ-adjusted GABA+ levels, and subclinical psychotic experiences using partial least squares structural equation modeling (PLS-SEM), which is a non-parametric analysis technique. All subjects enrolled in the ANOVA study were included. Basically, in this analysis, paths from bullying victimization to GABA+ levels and paths from GABA+ levels and subclinical psychotic experiences were regarded as the main connections, and the moderating effects of help-seeking intention on paths from bullying victimization to GABA+ levels were also investigated. We created a time lagged model, where latent variables at two time points were separately included, and a latent change score model, where latent baseline (intercept) variables and latent change (slope) variables were included. The PLS-SEM analyses were implemented using the SmartPLS 4.0 software, which allows us to estimate direct and moderating (interaction) effects. Bootstrapping method with 5,000 random resamples was used. The fit of the PLS-SEM model was evaluated using standardized root mean residual (SRMR), which is provided by the SmartPLS 4.0 software. An

SRMR value of less than 0.10 indicates an acceptable fit to the model.[16] Indirect effects were also assessed using PLE-SEM models.

Supplementary Result 1: Analysis of GABA+ levels

Associations over time between GABA+ levels and subclinical psychotic experiences

Associations over time between GABA+ levels and subclinical psychotic experiences were investigated. Subclinical psychotic experiences were not significantly associated with GABA+ levels at any time points (Time 1, n = 219, rho = -7.6×10^{-4}, FDR-corrected p = 0.99, uncorrected p = 0.99; Time 2, n = 211, rho = −0.035, FDR-corrected p = 0.93, uncorrected p = 0.62) (**Supplementary Fig. 4a,b**), and changes in subclinical psychotic experiences over time were not significantly associated with GABA+ level changes (n = 157, rho = −0.065, FDR-corrected p = 0.93, uncorrected p = 0.43) (**Supplementary Fig. 4c**).

Effects of bullying victimization and help-seeking intention on GABA+ levels

The effects of bullying victimization and help-seeking intention on GABA+ levels were investigated. All the assumptions of an ANOVA, such as the normal distribution of age-sex-SES-IQ-adjusted GABA+ levels, were met. There were no significant effects of bullying victimization (F = 2.0, p = 0.16), help-seeking intention (F = 0.99, p = 0.32), bullying victimization × help-seeking intention interaction (F = 0.65, p = 0.42), time (F = 0.044, p = 0.83), time × bullying victimization interaction (F = 0.82, p = 0.37), time × help-seeking intention interaction (F = 1.2, p = 0.28), or time × bullying victimization × help-seeking intention interaction (F = 0.038, p = 0.85) (n = 156) (**Supplementary Fig. 5**).

PLS-SEM analysis

Path analyses were performed to determine the relationships among variables including bullying victimization, help-seeking intention, age-sex-SES-IQ-adjusted GABA+ levels, and subclinical psychotic experiences using PLS-SEM. First, a time lagged model was created, and the PLS-SEM test revealed that the current model fitted the data well (n = 156, SRMR = 0.000) (**Supplementary Fig. 6a**). Specifically, the path coefficient (PC) from subclinical psychotic experiences at Time 1 to subclinical psychotic experiences at Time 2 was significant (PC = 0.32, p = 0.000), whereas the PC from bullying victimization to GABA+ levels at Time 1 (PC = −0.47, p = 0.45), PC from bullying victimization to GABA+ levels at Time 2 (PC = −0.23, p = 0.60), PC from GABA+ levels at Time 1 to GABA+ levels at Time 2 (PC = 0.094, p = 0.24), PC from GABA+ levels at Time 1 to subclinical psychotic experiences at Time 1 (PC = 0.064, p = 0.47), PC from GABA+ levels at Time 1 to subclinical psychotic experiences at Time 2 (PC = 0.021, p = 0.81), and PC from GABA+ levels at Time 2 to subclinical psychotic experiences at Time 2 were non-significant (PC = −0.11, p = 0.10). In addition, help-seeking intention did not significantly moderate the path from bullying victimization to GABA+ levels at Time 1 (PC =

0.21, *p* = 0.75) or to GABA+ levels at Time 2 (PC = 0.32, *p* = 0.49). No total indirect effects or specific indirect effects were found.

Next, a latent change score model was created, while the PLS-SEM test revealed that the current model fitted the data well (n = 156, SRMR = 0.085) (**Supplementary Fig. 6b**). Specifically, the PC from baseline GABA+ levels to differences in GABA+ levels (PC = 1.0, *p* = 0.000) and PC from baseline subclinical psychotic experiences levels to differences in subclinical psychotic experiences levels (PC = 0.97, *p* = 0.000) were significant, whereas PC from bullying victimization to baseline GABA+ levels (PC = −0.31, *p* = 0.50), PC from bullying victimization to differences in GABA+ levels (PC = 0.039, *p* = 0.63), PC from baseline GABA+ levels to baseline subclinical psychotic experiences (PC = −0.057, *p* = 0.43), PC from baseline GABA+ levels to differences in subclinical psychotic experiences (PC = −0.10, *p* = 1.0), and PC from differences in GABA+ levels to differences in subclinical psychotic experiences were non-significant (PC = 0.069, *p* = 1.0). In addition, help-seeking intention did not significantly moderate the path from bullying victimization to baseline GABA+ levels (PC = 0.36, *p* = 0.46) or the path from bullying victimization to differences in GABA+ levels (PC = −0.013, *p* = 0.86). No total indirect effects or specific indirect effects were found.

Supplementary Table 1: Flow chart of subject inclusion and exclusion

Association analysis / Exclusion criteria	Glx Time 1 – SPE Time 1	Glx Time 2 – SPE Time 2	Glx change – SPE change
	253 (No. of MRS data)	237 (No. of MRS data)	178 (No. of MRS data)
	↓ ↘	↓ ↘	↓ ↘
Abnormal brain organic findings (yes/no)	28 225	8 229	3 175
	↓ ↘	↓ ↘	↓ ↘
Glx CRLB >= 20% (yes/no)	2 223	0 229	2 173
	↓ ↘	↓ ↘	↓ ↘
Failure in coregistration/ segmentation (yes/no)	1 222	1 228	0 173
	↓ ↘	↓ ↘	↓ ↘
Missing SPE data (yes/no)	3 219	17 211	16 157

Abbreviations: Glx, combined glutamate-glutamine; SPE, subclinical psychotic experience; MRS, magnetic resonance spectroscopy; CRLB, Cramer-Rao lower bounds.

Supplementary Table 2: The association between bullying victimization and help-seeking intention

Time 1 data		BV		
$\chi2 = 1.5$, $p = 0.15$		Negative	Positive	Total
HSI	Negative	26	15	41
	Positive	128	47	175
	Total	154	62	216
Time 2 data		BV		
$\chi2 = 0.77$, $p = 0.25$		Negative	Positive	Total
HSI	Negative	24	12	36
	Positive	127	45	172
	Total	151	57	208
Times 1 and 2 data		BV		
$\chi2 = 0.84$, $p = 0.35$		Negative	Positive	Total
HSI	Negative	18	9	27
	Positive	97	32	129
	Total	115	41	156

Abbreviations: BV, bullying victimization; HSI, help-seeking intention.

Supplementary Fig. 1: Tissue segmentation of MRS VOI of a representative participant

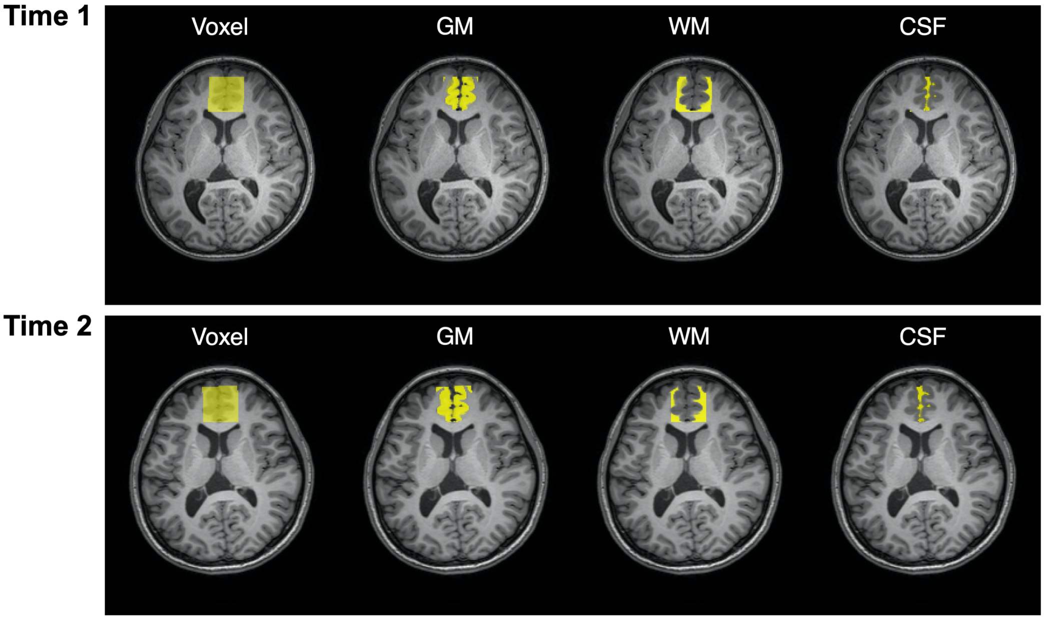

Magnetic resonance spectroscopy (MRS) data were processed to quantify tissue fractions using the Gannet 3.2 toolbox.[17] GannetCoRegister registers an MRS voxel-of-interest (VOI) to a T1-weighted image. GannetSegment segments a T1-weighted image using Statistical Parametric Mapping 12 (SPM12, www.fil.ion.ucl.ac.uk/spm) and quantifies the tissue fraction of gray matter (GM), white matter (WM) and cerebrospinal fluid (CSF) for the VOI. The images shown here are the data of the same subject as in **Fig. 1**. Abbreviations: GM, gray matter; WM, white matter; CSF, cerebrospinal fluid.

Supplementary Fig. 2: An example of failed coregistration/segmentation of MRS VOI

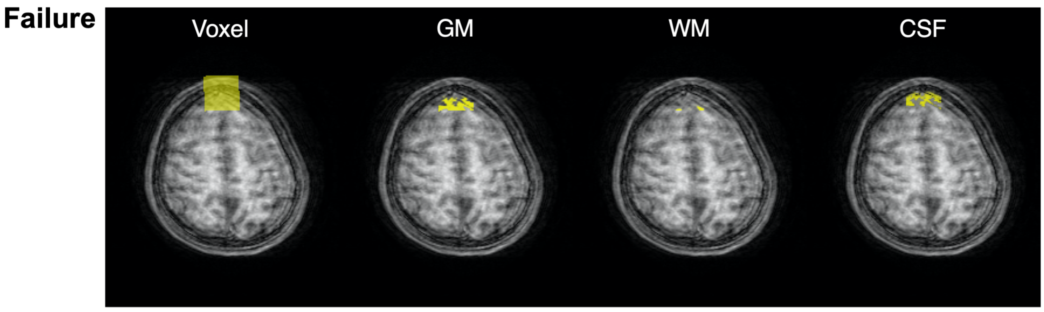

Example of the failure of coregistration/segmentation of an MRS VOI. This failure is assumed to be due to the low quality of the T1-weighted anatomical image (motion artifacts). Abbreviations: GM, gray matter; WM, white matter; CSF, cerebrospinal fluid.

Supplementary Fig. 3: Spectra fitting for GABA analysis

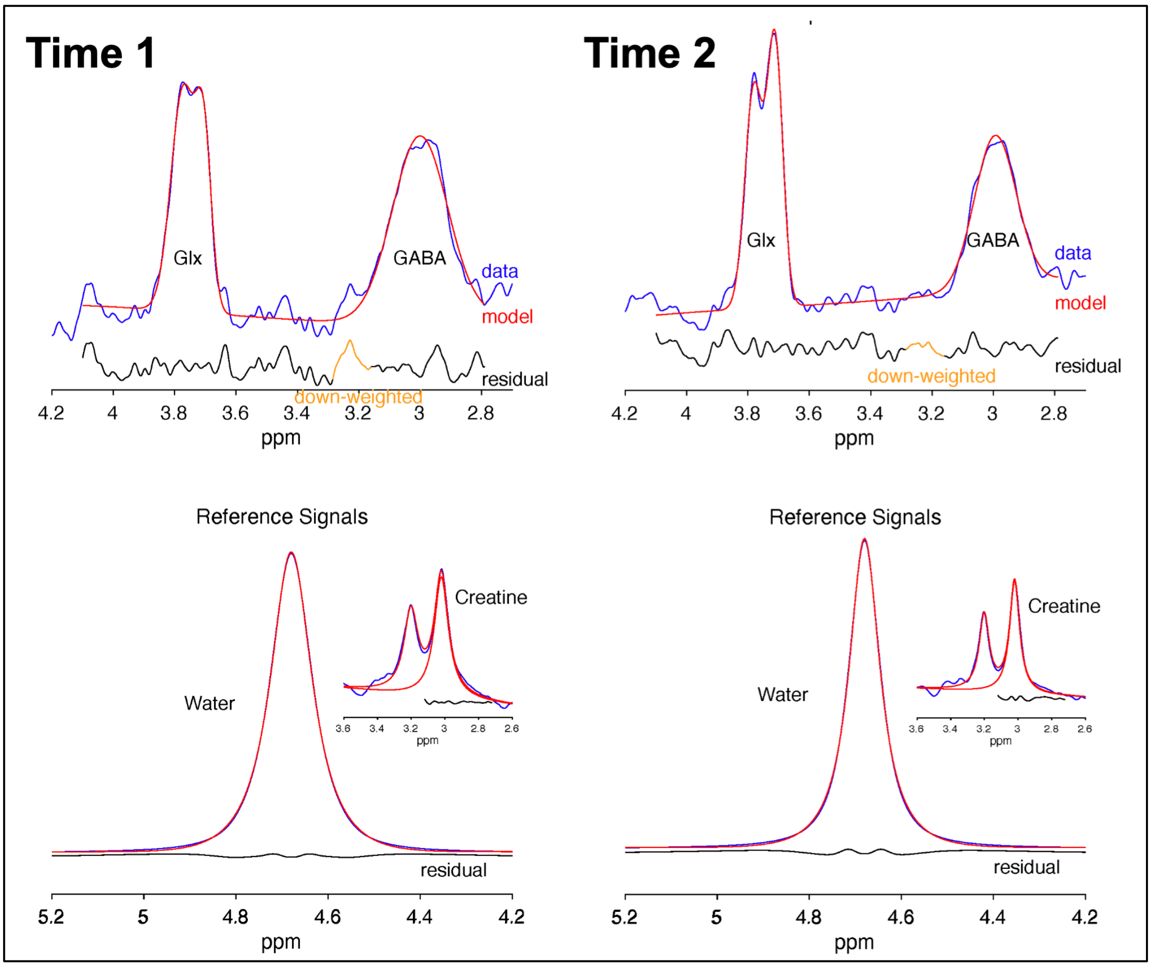

MEGA-editing was achieved with 15-ms Gaussian editing pulses applied at 1.90 ppm (ON) and 7.46 ppm (OFF) in alternate spectral lines. The actual spectra (the difference between edit ON and OFF spectra) (blue line) and their Gannet fits (red line) are displayed, where the edited GABA signal peak (~3 ppm) is clearly detectable. The data shown here belong to the same subject as in **Fig. 1** and **Supplementary Fig. 1**. Abbreviations: Glx, combined glutamate-glutamine; GABA, gamma-aminobutyric acid; ppm, parts per million.

Supplementary Fig. 4: Associations over time between GABA+ levels and signs of subclinical psychotic experiences

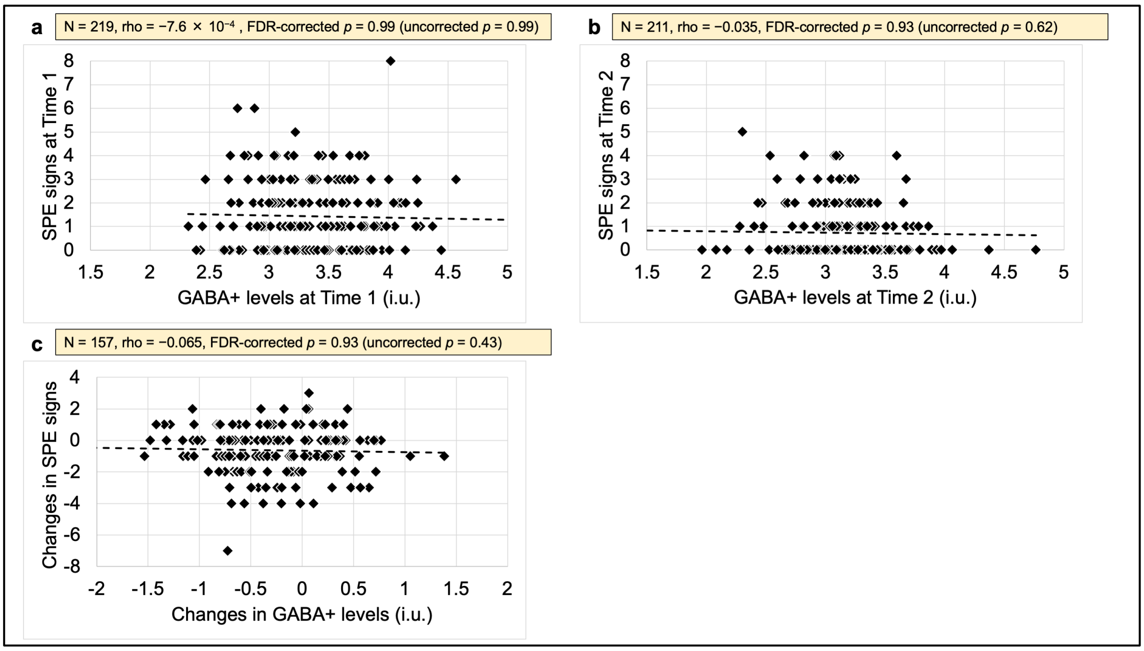

(**a**) The association at Time 1, (**b**) the association at Time 2, and (**c**) the association between longitudinal changes are illustrated. There were no significant associations between GABA+ levels in the pregenual ACC and SPEs at either Time 1 (**a**) or Time 2 (**b**). In addition, there was no significant association between over-time changes in the two variables (**c**). Abbreviations: GABA+, gamma-aminobutyric acid plus macromolecule; SPE, subclinical psychotic experience; i.u., institutional units.

Supplementary Fig. 5: Effects of bullying victimization and help-seeking intention on longitudinal GABA+ levels

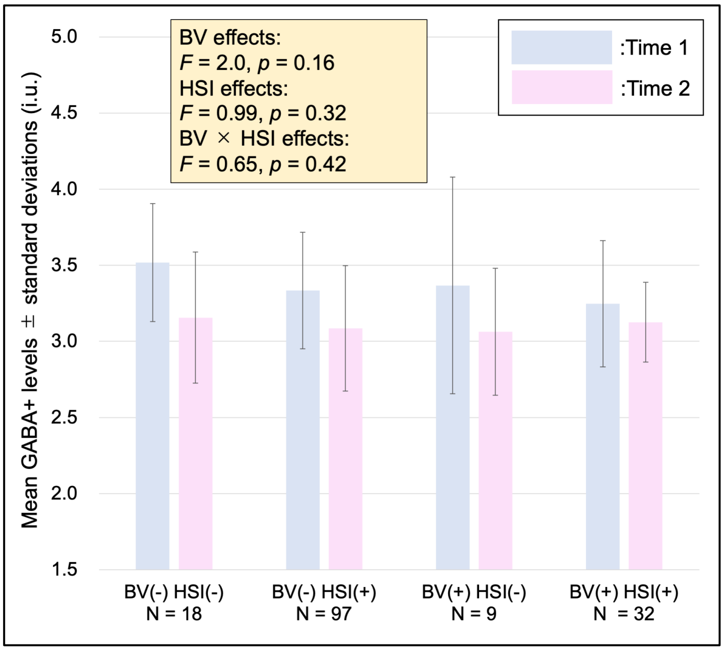

Light blue-colored bars represent Glx levels at Time 1, and pink-colored bars represent Glx levels at Time 2. Neither BV, HSI, nor BV × HSI interaction had significant effects on Glx levels. Abbreviations: BV, bullying victimization; HSI, help-seeking intention; GABA+, gamma-aminobutyric acid plus macromolecule; i.u., institutional units.

Supplementary Fig. 6: Results of PLS-SEM analysis for the associations among bullying victimization, help-seeking intention, GABA+, and subclinical psychotic experiences

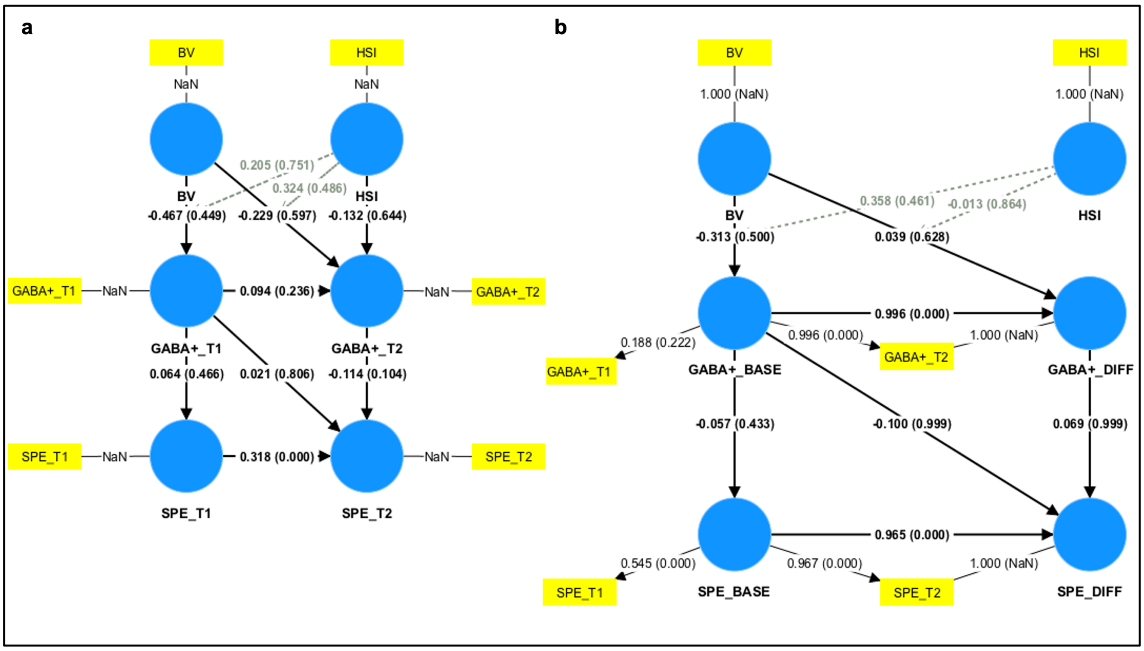

Path analyses were performed to determine the relationships among variables including BV, HSI, age-sex-SES-IQ-adjusted GABA+ levels, and SPEs using PLS-SEM. Results of **(a)** the time lagged model and of **(b)** the latent change score model are illustrated here. Solid arrows represent direct effects and dashed arrows represent moderation effects. Path coefficients (*p* values in parentheses) are shown on corresponding arrows. Indirect effects were also assessed, but no statistical significance was observed. Abbreviations: BV, bullying victimization; HSI, help-seeking intention; GABA+, gamma-aminobutyric acid plus macromolecule; T1, Time 1; T2, Time 2; SPE, subclinical psychotic experience; BASE, baseline; DIFF, difference.

Supplementary References

1. Okada N, Yahata N, Koshiyama D, Morita K, Sawada K, Kanata S *et al.* Neurometabolic and functional connectivity basis of prosocial behavior in early adolescence. *Sci Rep* 2019; **9:** 732.
2. Okada N, Yahata N, Koshiyama D, Morita K, Sawada K, Kanata S *et al.* Neurometabolic underpinning of the intergenerational transmission of prosociality. *Neuroimage* 2020; **218:** 116965.
3. Okada N, Ando S, Sanada M, Hirata-Mogi S, Iijima Y, Sugiyama H *et al.* Population-neuroscience study of the Tokyo TEEN Cohort (pn-TTC): Cohort longitudinal study to explore the neurobiological substrates of adolescent psychological and behavioral development. *Psychiatry Clin Neurosci* 2019; **73:** 231-242.
4. Ando S, Nishida A, Yamasaki S, Koike S, Morimoto Y, Hoshino A *et al.* Cohort Profile: The Tokyo Teen Cohort study (TTC). *Int J Epidemiol* 2019; **48:** 1414-1414g.
5. Poulton R, Caspi A, Moffitt TE, Cannon M, Murray R, Harrington H. Children's self-reported psychotic symptoms and adult schizophreniform disorder: a 15-year longitudinal study. *Arch Gen Psychiatry* 2000; **57:** 1053-1058.
6. Sijtsma H, Lee NC, Hollarek M, Walsh RJ, van Buuren M, Braams BR *et al.* Social cognition and friendships in adolescents with autistic-like experiences and psychotic-like experiences. *Front Psychiatry* 2020; **11:** 589824.
7. Jacobson S, Kelleher I, Harley M, Murtagh A, Clarke M, Blanchard M *et al.* Structural and functional brain correlates of subclinical psychotic symptoms in 11-13 year old schoolchildren. *Neuroimage* 2010; **49:** 1875-1885.
8. Jacobson McEwen SC, Connolly CG, Kelly AM, Kelleher I, O'Hanlon E, Clarke M *et al.* Resting-state connectivity deficits associated with impaired inhibitory control in non-treatment-seeking adolescents with psychotic symptoms. *Acta Psychiatr Scand* 2014; **129:** 134-142.
9. Okada N, Yahata N, Koshiyama D, Morita K, Sawada K, Kanata S *et al.* Abnormal asymmetries in subcortical brain volume in early adolescents with subclinical psychotic experiences. *Transl Psychiatry* 2018; **8:** 254.
10. Solberg ME, Olweus D. Prevalence estimation of school bullying with the Olweus Bully/Victim Questionnaire. *Aggress Behav* 2003; **29:** 239-268.
11. Fujikawa S, Ando S, Nishida A, Usami S, Koike S, Yamasaki S *et al.* Disciplinary slapping is associated with bullying involvement regardless of warm parenting in early adolescence. *J Adolesc* 2018; **68:** 207-216.

12. Ando S, Nishida A, Usami S, Koike S, Yamasaki S, Kanata S *et al.* Help-seeking intention for depression in early adolescents: Associated factors and sex differences. *J Affect Disord* 2018; **238:** 359-365.

13. Jorm AF, Kelly CM, Wright A, Parslow RA, Harris MG, McGorry PD. Belief in dealing with depression alone: results from community surveys of adolescents and adults. *J Affect Disord* 2006; **96:** 59-65.

14. Inada N, Kamio Y. Short forms of the Japanese version WISC-III for assessment of children with autism spectrum disorders. *Jpn J Child Adolesc Psychiatry* 2010; **51:** 11-19.

15. Faul F, Erdfelder E, Lang AG, Buchner A. G*Power 3: a flexible statistical power analysis program for the social, behavioral, and biomedical sciences. *Behav Res Methods* 2007; **39:** 175-191.

16. Kline RB. *Principles and Practice of Structural Equation Modeling, 4th ed.*, The Guilford Press: New York, 2016.

17. Edden RA, Puts NA, Harris AD, Barker PB, Evans CJ. Gannet: A batch-processing tool for the quantitative analysis of gamma-aminobutyric acid–edited MR spectroscopy spectra. *J Magn Reson Imaging* 2014; **40:** 1445-1452.
